# Correction: Clinical efficacy of different methods for treatment of granulomatous lobular mastitis: A systematic review and network meta-analysis

**DOI:** 10.1371/journal.pone.0331152

**Published:** 2025-08-28

**Authors:** Yunxiang Zhou, Leilai Xu

The first author’s name is spelled incorrectly. The correct name is: Yunxiang Zhou. The correct citation is: Zhou Y, Xu L (2025) Clinical efficacy of different methods for treatment of granulomatous lobular mastitis: A systematic review and network meta-analysis. PLoS ONE 20(2): e0318236. https://doi.org/10.1371/journal.pone.0318236
