# Supplementary material for: Clinical efficacy of different methods for treatment of granulomatous lobular mastitis: A systematic review and network meta-analysis
Source: PLoS One. 2025 Feb 3;20(2):e0318236. doi: 10.1371/journal.pone.0318236 (PMC11790104; doi:10.1371/journal.pone.0318236)
Supplement: S1 Table — (DOCX) [file pone.0318236.s002.docx]

| **Study** | **Year** | **Inclusion /exclusion** | **Reason for exclusion** |
| --- | --- | --- | --- |
| Author 001 | 2022 | Inclusion | / |
| Author 002 | 2019 | Inclusion | / |
| Author 003 | 2021 | Inclusion | / |
| Author 004 | 2015 | Inclusion | / |
| Author 005 | 2013 | Inclusion | / |
| Author 006 | 2018 | Inclusion | / |
| Author 007 | 2021 | Inclusion | / |
| Author 008 | 2014 | Inclusion | / |
| Author 009 | 2013 | Inclusion | / |
| Author 010 | 2014 | Inclusion | / |
| Author 011 | 2005 | Inclusion | / |
| Author 012 | 2020 | Inclusion | / |
| Author 013 | 2020 | Inclusion | / |
| Author 014 | 2013 | Inclusion | / |
| Author 015 | 2013 | Inclusion | / |
| Author 016 | 2011 | Inclusion | / |
| Author 017 | 2015 | Inclusion | / |
| Author 018 | 2017 | Inclusion | / |
| Author 019 | 2014 | Inclusion | / |
| Author 020 | 2022 | exclusion | Does not conform to the PICOS criteria |
| Author 021 | 2006 | exclusion | Does not conform to the PICOS criteria |
| Author 022 | 2014 | exclusion | Does not conform to the PICOS criteria |
| Author 023 | 2019 | exclusion | Does not conform to the PICOS criteria |
| Author 024 | 2021 | exclusion | The article type is a letter and does not conform to the PICOS criteria. |
| Author 025 | 2020 | exclusion | The article type is a Protocol and does not conform to the PICOS criteria. |
| Author 026 | 2022 | exclusion | Does not conform to the PICOS criteria |
| Author 027 | 2022 | exclusion | The article type is a Review and does not conform to the PICOS criteria. |
| Author 028 | 2022 | exclusion | The article type is a Protocol and does not conform to the PICOS criteria. |
| Author 029 | 2003 | exclusion | This article only analyzes the risk factors associated with granulomatous mastitis and does not compare treatment methods. |
| Author 030 | 2020 | exclusion | There is only one treatment plan, lacking comparison. |
| Author 031 | 2014 | exclusion | "There is no comparison of treatment methods." |
| Author 032 | 2015 | exclusion | There is only one treatment plan, lacking comparison. |
| Author 033 | 2014 | exclusion | There is only one treatment plan, lacking comparison. |
| Author 034 | 2020 | exclusion | The study subjects are patients with benign fibromas. |
