# Supplementary material for: Clinical efficacy of different methods for treatment of granulomatous lobular mastitis: A systematic review and network meta-analysis
Source: PLoS One. 2025 Feb 3;20(2):e0318236. doi: 10.1371/journal.pone.0318236 (PMC11790104; doi:10.1371/journal.pone.0318236)
Supplement: S2 Table — (DOCX) [file pone.0318236.s003.docx]

| **Study** | **Data extractors/ Date** | **Intervention** | **Sample size** | **Total recurrence** | **Inclusion /exclusion** |
| --- | --- | --- | --- | --- | --- |
| Author 001 2022 | Extractor 001 / May 2024 | Steroid Therapy | 16 | 2 | Inclusion |
|  | Extractor 001 / May 2024 | Local steroid injection | 42 | 2 | Inclusion |
| Author 002 2019 | Extractor 001 / May 2024 | Steroid Therapy | 44 | 10 | Inclusion |
|  | Extractor 001 / May 2024 | Surgery+Steroid Therapy | 156 | 8 | Inclusion |
| Author 003 2019[33] | Extractor 001 / May 2024 | Local steroid injection | 38 | 0 | Inclusion |
|  | Extractor 001 / May 2024 | Surgery | 48 | 15 | Inclusion |
| Author 004 2021[32] | Extractor 001 / May 2024 | Steroid Therapy | 44 | 9 | Inclusion |
|  | Extractor 001 / May 2024 | Surgery | 33 | 0 | Inclusion |
| Author 005  2013[17] | Extractor 002 / May 2024 | Steroid Therapy | 10 | 0 | Inclusion |
|  | Extractor 002 / May 2024 | Surgery | 2 | 2 | Inclusion |
| Author 006 2018[31] | Extractor 002 / May 2024 | Steroid Therapy | 6 | 1 | Inclusion |
|  | Extractor 002 / May 2024 | Surgery | 30 | 4 | Inclusion |
| Author 007  2021[30] | Extractor 001 / May 2024 | Steroid Therapy | 23 | 9 | Inclusion |
|  | Extractor 001 / May 2024 | Surgery | 17 | 7 | Inclusion |
|  | Extractor 001 / May 2024 | Steroid Therapy+ drainage | 47 | 9 | Inclusion |
| Author 008  2014[29] | Extractor 002 / May 2024 | Surgery | 53 | 4 | Inclusion |
|  | Extractor 002 / May 2024 | Surgery+Steroid Therapy | 21 | 0 | Inclusion |
| Author 009 2013[28] | Extractor 002 / May 2024 | Observation | 8 | 0 | Inclusion |
|  | Extractor 002 / May 2024 | Steroid Therapy | 13 | 1 | Inclusion |
|  | Extractor 002 / May 2024 | Surgery | 23 | 2 | Inclusion |
|  | Extractor 002 / May 2024 | Drainage | 14 | 1 | Inclusion |
| Author 010  2014[27] | Extractor 002 / May 2024 | Steroid Therapy | 23 | 7 | Inclusion |
|  | Extractor 002 / May 2024 | Surgery+Steroid Therapy | 37 | 0 | Inclusion |
| Author 011  2005[18] | Extractor 001 / May 2024 | Observation | 4 | 0 | Inclusion |
|  | Extractor 001 / May 2024 | Surgery | 1 | 0 | Inclusion |
| Author 012  2020[26] | Extractor 001 / May 2024 | Surgery | 50 | 1 | Inclusion |
|  | Extractor 001 / May 2024 | Surgery+Traditional Chinese Medicine | 60 | 0 | Inclusion |
| Author 013 2020[25] | Extractor 002 / May 2024 | Surgery | 25 | 4 | Inclusion |
|  | Extractor 002 / May 2024 | Surgery+Traditional Chinese Medicine | 28 | 0 | Inclusion |
| Author 014  2013[24] | Extractor 002 / May 2024 | Surgery+Steroid Therapy | 28 | 6 | Inclusion |
|  | Extractor 002 / May 2024 | Surgery+Local steroid injection+ Steroid Therapy | 34 | 5 | Inclusion |
| Author 015 2013[23] | Extractor 002 / May 2024 | Steroid Therapy | 25 | 5 | Inclusion |
|  | Extractor 002 / May 2024 | Surgery | 18 | 3 | Inclusion |
| Author 016  2011[22] | Extractor 001 / May 2024 | Steroid Therapy | 5 | 0 | Inclusion |
|  | Extractor 001 / May 2024 | Steroid Therapy+Antibiotics | 2 | 0 | Inclusion |
| Author 017  2015[21] | Extractor 002 / May 2024 | Steroid Therapy | 15 | 3 | Inclusion |
|  | Extractor 002 / May 2024 | Steroid Therapy+MTX | 6 | 0 | Inclusion |
| Author 018 2017[20] | Extractor 001 / May 2024 | Surgery | 20 | 5 | Inclusion |
|  | Extractor 001 / May 2024 | Steroid Therapy+ drainage | 14 | 1 | Inclusion |
| Author 019  2014[19] | Extractor 001 / May 2024 | Surgery | 2 | 0 | Inclusion |
|  | Extractor 001 / May 2024 | Antibiotics | 7 | 3 | Inclusion |
|  | Extractor 001 / May 2024 | Antibiotics+drainage | 3 | 2 | Inclusion |
