# Supplementary material for: Clinical efficacy of different methods for treatment of granulomatous lobular mastitis: A systematic review and network meta-analysis
Source: PLoS One. 2025 Feb 3;20(2):e0318236. doi: 10.1371/journal.pone.0318236 (PMC11790104; doi:10.1371/journal.pone.0318236)
Supplement: S3 Table — (DOCX) [file pone.0318236.s004.docx]

| **(NOS) Checklist** | **Selection** | | | | **Comparability** | **Exposure** | | | **Quality assessment** |
| --- | --- | --- | --- | --- | --- | --- | --- | --- | --- |
| **Study** | **Case definition adequate** | **Representativeness of cases** | **Selection of controls** | **Definition of controls** | **Comparability of cohorts** | **Ascertainment of exposure** | **Same method of ascertainment for groups** | **Non-response rate** | **Quality assessment** |
| Author 003 2022[35] | 1 | 1 | 1 | 1 | 2 | 0 | 1 | 1 | 8/9 |
| Author 004 2019[34] | 1 | 1 | 1 | 0 | 2 | 1 | 1 | 1 | 8/9 |
| Author 005 2021[33] | 1 | 1 | 1 | 0 | 1 | 1 | 1 | 1 | 7/9 |
| Author 006 2015[32] | 1 | 1 | 1 | 0 | 2 | 1 | 1 | 1 | 8/9 |
| Author 007 2013[17] | 1 | 1 | 1 | 0 | 2 | 0 | 1 | 1 | 7/9 |
| Author 008 | 1 | 1 | 1 | 0 | 2 | 0 | 1 | 1 | 7/9 |
| Author 009 2021[30] | 1 | 1 | 1 | 1 | 1 | 0 | 1 | 1 | 7/9 |
| Author 010 2014[29] | 1 | 1 | 1 | 0 | 2 | 0 | 1 | 0 | 6/9 |
| Author 011 2013[28] | 1 | 1 | 1 | 0 | 1 | 0 | 1 | 1 | 6/9 |
| Author 012 2014[27] | 1 | 1 | 1 | 1 | 1 | 0 | 1 | 1 | 7/9 |
| Author 013 2005[18] | 1 | 1 | 1 | 0 | 1 | 0 | 1 | 1 | 6/9 |
| Author 014 2020[26] | 1 | 1 | 1 | 1 | 1 | 1 | 1 | 0 | 7/9 |
| Author 015 | 1 | 1 | 1 | 0 | 2 | 1 | 1 | 0 | 7/9 |
| Author 016 2013[24] | 1 | 1 | 1 | 0 | 2 | 0 | 1 | 1 | 7/9 |
| Author 017 | 1 | 1 | 1 | 0 | 2 | 0 | 1 | 1 | 7/9 |
| Author 018 2011[22] | 1 | 1 | 1 | 0 | 2 | 0 | 1 | 0 | 6/9 |
| Author 019 2015[21] | 1 | 1 | 1 | 0 | 2 | 0 | 1 | 1 | 7/9 |
| Author 020 | 1 | 1 | 1 | 1 | 2 | 0 | 1 | 1 | 8/9 |
| Author 021 [19] | 1 | 1 | 1 | 0 | 1 | 0 | 1 | 1 | 6/9 |
