# Supplementary material for: Clinical efficacy of different methods for treatment of granulomatous lobular mastitis: A systematic review and network meta-analysis
Source: PLoS One. 2025 Feb 3;20(2):e0318236. doi: 10.1371/journal.pone.0318236 (PMC11790104; doi:10.1371/journal.pone.0318236)
Supplement: Attachment — Submitted filename: PONE-D-24-22261 (1).pdf [file pone.0318236.s005.pdf]

PLOS ONE

Clinical efficacy of different methods for treatment of granulomatous lobular mastitis: a systematic review and network meta-analysis

--Manuscript Draft--

**Manuscript Number:**	PONE-D-24-22261
**Article Type:**	Research Article
**Full Title:**	Clinical efficacy of different methods for treatment of granulomatous lobular mastitis: a systematic review and network meta-analysis
**Short Title:**	Clinical efficacy of different methods for treatment of granulomatous lobular mastitis
**Corresponding Author:**	LEILAI XU<br>zhejiang zhongyiyao daxue diyi linchuang xueyuan: Zhejiang Chinese Medical University First Clinical Medical College<br>Hangzhou, zhejiang CHINA
**Keywords:**	granulomatous lobular mastitis, recurrence, network meta-analysis, treatment
**Abstract:**	Objective<br>This network meta-analysis aims to evaluate the recurrence rates of various treatment options for granulomatous lobular mastitis.<br>Methods<br>We systematically searched and identified eligible studies in PubMed, EMBASE, Cochrane Library, and Web of Science databases until September 30, 2023. Original studies reporting the recurrence rates of various treatments were included. Subsequently, literature screening, data extraction, and network meta-analysis were conducted. This study was registered with PROSPERO (registration number CRD 42023434773).<br>Results<br>Nineteen articles involving 1,095 patients were included in this study. The network meta-analysis revealed that several treatment combinations reduced the recurrence rate compared to observation: Surgery + Local steroid injection + Systemic steroids therapy (OR: 0.23, 95% CI 0.01 to 4.53), Local steroid injection (OR: 0.34, 95% CI 0.02 to 6.81), Surgery + Systemic steroids therapy (OR: 0.36, 95% CI 0.02 to 5.29), Surgery + Traditional Chinese Medicine (OR: 0.33, 95% CI 0.01 to 9.11), Systemic steroids therapy + MTX (OR: 0.62, 95% CI 0.01 to 34.59), and Systemic steroids therapy + drainage (OR: 0.76, 95% CI 0.05 to 10.67). Among these, Surgery + Local steroid injection + Systemic steroids therapy demonstrated superior efficacy. The surface under cumulative ranking curve (SUCRA) values were highest for Surgery + Local steroid injection + Systemic steroids therapy (0.85), followed by Local steroid injection (0.78) and Surgery + Systemic steroids therapy (0.77).<br>Conclusions<br>Steroid-based combination therapy may be the first choice for IGM patients, with a comprehensive strategy of local and systemic steroids combined with surgery having the best effect on IGM.
**Order of Authors:**	Yuxiang Zhou
	LEILAI XU
**Additional Information:**	
**Question**	**Response**
**Financial Disclosure**<br><br>Enter a financial disclosure statement that describes the sources of funding for the work included in this submission. Review the submission guidelines for detailed requirements. View published research articles from *PLOS ONE* for specific	The author(s) received no specific funding for this work.

examples.<br><br>This statement is required for submission and **will appear in the published article** if the submission is accepted. Please make sure it is accurate.<br><br>**Funded studies**<br>Enter a statement with the following details:<br>• Initials of the authors who received each award<br>• Grant numbers awarded to each author<br>• The full name of each funder<br>• URL of each funder website<br>• Did the sponsors or funders play any role in the study design, data collection and analysis, decision to publish, or preparation of the manuscript?<br><br>Did you receive funding for this work?	
**Competing Interests**<br><br>Use the instructions below to enter a competing interest statement for this submission. On behalf of all authors, disclose any competing interests that could be perceived to bias this work—acknowledging all financial support and any other relevant financial or non-financial competing interests.<br><br>This statement is required for submission and **will appear in the published article** if the submission is accepted. Please make sure it is accurate and that any funding sources listed in your Funding Information later in the submission form are also declared in your Financial Disclosure statement.<br><br>View published research articles from *PLOS ONE* for specific examples.	The authors have declared that no competing interests exist.

**NO authors have competing interests**<br><br>Enter: *The authors have declared that no competing interests exist.*<br><br>**Authors with competing interests**<br><br>Enter competing interest details beginning with this statement:<br><br>*I have read the journal's policy and the authors of this manuscript have the following competing interests: [insert competing interests here]*<br><br>* typeset	
**Ethics Statement**<br><br>Enter an ethics statement for this submission. This statement is required if the study involved:<br><br>• Human participants<br>• Human specimens or tissue<br>• Vertebrate animals or cephalopods<br>• Vertebrate embryos or tissues<br>• Field research<br><br>Write "N/A" if the submission does not require an ethics statement.<br><br>General guidance is provided below. Consult the submission guidelines for detailed instructions. **Make sure that all information entered here is included in the Methods section of the manuscript.**	N/A

Powered by Editorial Manager® and ProduXion Manager® from Aries Systems Corporation

**Format for specific study types**<br><br>**Human Subject Research (involving human participants and/or tissue)**<br>• Give the name of the institutional review board or ethics committee that approved the study<br>• Include the approval number and/or a statement indicating approval of this research<br>• Indicate the form of consent obtained (written/oral) or the reason that consent was not obtained (e.g. the data were analyzed anonymously)<br><br>**Animal Research (involving vertebrate animals, embryos or tissues)**<br>• Provide the name of the Institutional Animal Care and Use Committee (IACUC) or other relevant ethics board that reviewed the study protocol, and indicate whether they approved this research or granted a formal waiver of ethical approval<br>• Include an approval number if one was obtained<br>• If the study involved *non-human primates*, add *additional details* about animal welfare and steps taken to ameliorate suffering<br>• If anesthesia, euthanasia, or any kind of animal sacrifice is part of the study, include briefly which substances and/or methods were applied<br><br>**Field Research**<br><br>Include the following details if this study involves the collection of plant, animal, or other materials from a natural setting:<br>• Field permit number<br>• Name of the institution or relevant body that granted permission	
**Data Availability**<br><br>Authors are required to make all data underlying the findings described fully available, without restriction, and from the time of publication. PLOS allows rare exceptions to address legal and ethical concerns. See the PLOS Data Policy and FAQ for detailed information.	No - some restrictions will apply

Powered by Editorial Manager® and ProduXion Manager® from Aries Systems Corporation

A Data Availability Statement describing where the data can be found is required at submission. Your answers to this question constitute the Data Availability Statement and **will be published in the article**, if accepted.<br><br>**Important:** Stating 'data available on request from the author' is not sufficient. If your data are only available upon request, select 'No' for the first question and explain your exceptional situation in the text box.<br><br>Do the authors confirm that all data underlying the findings described in their manuscript are fully available without restriction?	
**Describe where the data may be found in full sentences. If you are copying our sample text, replace any instances of XXX with the appropriate details.**<br><br>• If the data are **held or will be held in a public repository**, include URLs, accession numbers or DOIs. If this information will only be available after acceptance, indicate this by ticking the box below. For example: *All XXX files are available from the XXX database (accession number(s) XXX, XXX.).*<br>• If the data are all contained **within the manuscript and/or Supporting Information files**, enter the following: *All relevant data are within the manuscript and its Supporting Information files.*<br>• If neither of these applies but you are able to provide **details of access elsewhere**, with or without limitations, please do so. For example:<br><br>*Data cannot be shared publicly because of [XXX]. Data are available from the XXX Institutional Data Access / Ethics Committee (contact via XXX) for researchers who meet the criteria for access to confidential data.*<br><br>*The data underlying the results presented in the study are available from (include the name of the third party*	All relevant data are within the manuscript and its Supporting Information files.

and contact information or URL).<br>• This text is appropriate if the data are owned by a third party and authors do not have permission to share the data.<br><br>* typeset	
Additional data availability information:	Tick here if your circumstances are not covered by the questions above and you need the journal’s help to make your data available.

Manuscript

Click here to access/download;Manuscript;renamed_9685c.docx

Clinical efficacy of different methods for treatment of granulomatous lobular mastitis: a systematic review and network meta-analysis

Yuxiang Zhou[1,2], Leilai Xu[3]

1. Zhejiang Chinese Medical University, Hangzhou, China.
2. The First People's Hospital of Yuhang District, Hangzhou, China.
3. First Affiliated Hospital of Zhejiang Chinese Medical University Hangzhou, China.

Correspondence: Leilai Xu,MD

Department of Breast Surgery, First Affiliated Hospital of Zhejiang Chinese Medical University, Youdian Road 54#, Hangzhou, China (e-mail: xll1005@126.com).

Conflict of interest

No potential conflict of interest relevant to this article was reported.

Funding

This research was supported by a grant from the Zhejiang Traditional Chinese Medicine Administration (2023ZL416).

Author contributions

Data curation: Yuxiang Zhou ;**Formal analysis**: Yuxiang Zhou;

Methodology: Leilai Xu;**Resources:** Leilai Xu;**Software:** Leilai Xu;

Supervision: Leilai Xu ;**Writing – original draft:** Yuxiang Zhou;

Writing – review & editing: Leilai Xu

Abstract

Objective

This network meta-analysis aims to evaluate the recurrence rates of various treatment options for granulomatous lobular mastitis.

Methods

We systematically searched and identified eligible studies in PubMed, EMBASE, Cochrane Library, and Web of Science databases until September 30, 2023. Original studies reporting the recurrence rates of various treatments were included. Subsequently, literature screening, data extraction, and network meta-analysis were conducted. This study was registered with PROSPERO (registration number CRD 42023434773).

Results

Nineteen articles involving 1,095 patients were included in this study. The network meta-analysis revealed that several treatment combinations reduced the recurrence rate compared to observation: Surgery + Local steroid injection + Systemic steroids therapy (OR: 0.23, 95% CI 0.01 to 4.53), Local steroid injection (OR: 0.34, 95% CI 0.02 to 6.81), Surgery + Systemic steroids therapy (OR: 0.36, 95% CI 0.02 to 5.29), Surgery + Traditional Chinese Medicine (OR: 0.33, 95% CI 0.01 to 9.11), Systemic steroids therapy + MTX (OR: 0.62, 95% CI 0.01 to 34.59), and Systemic steroids therapy + drainage (OR: 0.76, 95% CI 0.05 to 10.67). Among these, Surgery + Local steroid injection + Systemic steroids therapy demonstrated superior efficacy. The surface under cumulative ranking curve (SUCRA) values were highest for Surgery + Local steroid injection + Systemic steroids therapy (0.85), followed by Local steroid injection (0.78)

and Surgery + Systemic steroids therapy (0.77).

Conclusions

Steroid-based combination therapy may be the first choice for IGM patients, with a comprehensive strategy of local and systemic steroids combined with surgery having the best effect on IGM.

Keywords: granulomatous lobular mastitis, recurrence, network meta-analysis, treatment

Introduction

Granulomatous mastitis, also known as Granulomatous lobular mastitis, is a benign chronic inflammatory condition of the breast tissue[1]. This condition was first described by Kessler and Wolloch in 1972. Granulomatous mastitis is characterized by the absence of an apparent underlying cause, making its diagnosis and management particularly challenging[2]. The condition predominantly affects women in their 30s and 40s, a demographic typically within the childbearing age range. It is most commonly observed within a few years following childbirth. It is particularly prevalent within a few years postpartum, highlighting a potential link with the physiological changes that occur during and after pregnancy. Despite its benign nature, the lack of a clear underlying cause makes granulomatous mastitis a condition that requires careful clinical attention to manage effectively[3].

Idiopathic granulomatous mastitis (IGM) is a rare, benign inflammatory condition characterized by noncaseating granulomatous inflammation within the breast lobules,

with possible microabscess formation. The diagnosis of IGM requires a multifaceted approach, integrating clinical, radiological, or sonographic evidence with histological examination to confirm the presence of characteristic granulomas[4, 5]. The etiology and pathogenesis of IGM remain unclear, making it a challenging condition to understand and manage. However, several factors are suspected to contribute to its development. These include α1-antitrypsin deficiency, smoking, Corynebacterium infection, hyperprolactinemia, use of oral contraceptives, and autoimmune abnormalities. Despite its benign nature, the lack of a clear underlying cause necessitates careful clinical attention to effectively diagnose and manage the condition[6-8].

Idiopathic granulomatous mastitis (IGM) poses a challenge in clinical management due to the absence of a standardized treatment regimen. Treatment options are diverse, including antibiotics, steroids, immunosuppressive therapies like methotrexate, surgical interventions such as wide local excision or mastectomy, traditional Chinese medicine, and observation. Clinician experience plays a pivotal role in the selection of treatment modalities. Effectiveness and recurrence rates vary across studies, contributing to the complexity of decision-making[5, 9-12].

Meta-analyses have been limited by the lack of direct comparisons among interventions[13, 14]. To address this issue, we employed a network meta-analysis approach to directly and indirectly evaluate and compare various interventions. This method provides valuable insights into the efficacy of different treatment options, offering a more informed basis for clinical decision-making in the management of IGM.

Materials and methods

Search strategy

This network meta-analysis adhered to the PRISMA-NMA guidelines to ensure a rigorous and systematic review of healthcare interventions[15]. A comprehensive search strategy was implemented, utilizing MeSH terms and keywords related to treatment, management, intervention, remission, recurrence, prognosis, idiopathic, mastitis, and granulomatous conditions. The search spanned several major databases, including PubMed, EMBASE, the Cochrane Library, and Web of Science, covering studies available up to September 30, 2023. Only English-language studies were included, focusing exclusively on human interventional studies. This methodical approach aimed to provide a thorough and precise synthesis of the current evidence base.

Study selection criteria

The study selection process was meticulously carried out by two independent reviewers, FSY and XLF, with any disagreements resolved through thorough discussion.

Inclusion Criteria:

Study Types: Included were published research articles encompassing randomized controlled trials, cohort studies, and case/control studies.

Population: Studies involving human clinical trials with various interventions for Idiopathic Granulomatous Mastitis (IGM).

Interventions: The interventions considered included oral steroids, topical steroids, methotrexate (MTX), drainage, and surgical management such as lumpectomy, wide

local excision, and mastectomy, as well as observation and prolactin-lowering agents, either alone or in combination.

Diagnosis: All included studies confirmed IGM diagnosis through histopathological examination.

Outcomes: Studies that reported recurrence rates of the different treatments for IGM.

Exclusion Criteria:

Publication Types: Conference abstracts, comments, letters, animal studies, reviews, case reports, ecological studies, cross-sectional studies, in vitro studies, and duplicate publications were excluded.

Data Reporting: Studies that did not report or calculate the desired parameters were excluded.

Data extraction and quality assessment

In this meta-analysis, a meticulous approach was undertaken for quality assessment and data extraction to uphold the highest methodological standards. Two independent investigators, FSY and XLF, conducted these processes to ensure objectivity and minimize bias. The data extraction checklist included critical elements such as the first author, publication year, patient number, recurrence rate, intervention details, sample size, follow-up period, and observation indices.

Quality assessment was performed using the Newcastle–Ottawa Quality Assessment Scale (NOS), a validated tool for evaluating cohort and case-control studies. The NOS criteria encompass patient selection, group comparability, and ascertainment of exposure and outcomes. Studies were classified according to their NOS scores: 7-8

points indicated very good quality, 5-6 points denoted good quality, 4 points were satisfactory, and scores of 0-3 points were considered unsatisfactory.

Data analysis

A network meta-analysis was conducted using odds ratio (OR) values to evaluate the recurrence rate of idiopathic granulomatous mastitis (IGM), with a 95% confidence interval (CI) for both indirect and mixed comparisons. The assessment included checks for similarity, transitivity, and consistency. Clinical evaluation was employed for transitivity, while consistency was formally assessed[16]. Global and local inconsistency were examined through a χ^2 test and side-splitting, respectively. Probability estimates for ranking each intervention were calculated, and comparison-adjusted funnel plots were used to evaluate publication bias. Additionally, sensitivity analysis was performed to assess the impact of individual studies, involving the exclusion of studies with a high risk of bias or those contributing to global or local inconsistency. Statistical evaluations of inconsistency and the creation of network graphs and figures were carried out using the network and network graphs packages in STATA version 15 (STATA Corporation, College Station, TX, USA). Following preliminary searches, the protocol was registered with PROSPERO (registration number CRD42023434773). All analyses were grounded in previously published studies, obviating the need for ethical approval and patient consent.

Results

Search results

The initial search of the databases yielded 573 articles. Following the elimination of

duplicates, 376 papers underwent title and abstract screening, resulting in the exclusion of 329 papers. Subsequently, 47 papers were assessed for eligibility through full-text review, with 19 studies ultimately included in the meta-analysis[17-35]. The PRISMA flow diagram illustrating the study selection process is presented in Fig. 1.

Eligible studies and patient characteristics

The basic characteristics of the eligible studies are outlined in Table 1. All studies included were published in English between 2013 and 2022. No randomized controlled trials were identified; the majority of the studies were retrospective. The most frequently employed interventions for IGM were steroids, surgical management, and a combination of steroids and surgical management. Most of the studies included had two arms, with only two studies having three or four arms. The risk of bias assessment for the 19 included trials are summarized in Table 1, evaluated using the Newcastle-Ottawa Scale (NOS) checklist. The median NOS quality score for cohort studies ranged from 6 to 7, indicating medium to high quality.

Network diagrams

The 19 included studies covered thirteen different treatments: Observation(Obs), Systemic steroids therapy(SST), Local steroid injection(LSI), Surgery(Sur), Surgery+ Systemic steroids therapy(SurSST), Systemic steroids therapy+drainage (SSTDra), drainage(Dra), Surgery+ Traditional Chinese Medicine(SurTCM), Surgery+Local steroid injection+Systemic steroids therapy (LSISSTSur), Systemic steroids therapy+ Antibiotics(SSTATB), Systemic steroids therapy+MTX(SSTMTX), Antibiotics(ATB), Antibiotics+drainage(ATBDra). The maximum sample size of treatment was Systemic

steroids therapy and Surgery, shown in **Fig. 2.** The thickness of the lines in the network graph is proportional to the number of studies included in each pairwise comparison, and the diameter of the circles is proportional to the number of participants who received each intervention.

Testing for inconsistency

The p-value for the test of overall inconsistency was greater than 0.05 (p = 0.9682). Similarly, no p-values were below 0.05 for the test of local inconsistency. Neither the global nor the local tests showed significance, indicating that the assumption of consistency was upheld.

Outcomes of the network meta-analysis

Network meta-analysis showed that, in comparison with observation, three-combined treatment (Surgery+Local steroid injection+ Systemic steroids therapy) ranked the best for the recurrence rate of different treatments for IGM reduced(OR: 0.23, 95%CI 0.01 to 4.53), followed by Local steroid injection(OR: 0.34, 95%CI 0.02 to 6.81), two-combined treatment (Surgery+Systemic steroids therapy)(OR: 0.36, 95%CI 0.02 to 5.29), (Surgery+ Traditional Chinese Medicine)(OR: 0.33, 95%CI 0.01 to 9.11), (Systemic steroids therapy+ MTX)(OR: 0.62, 95%CI 0.01 to 34.59), (Systemic steroids therapy+ drainage)(OR: 0.76, 95%CI 0.05 to 10.67), all of which ranked higher than observation(**Fig 3, Tables 2nd3**). However, drainage, systemic steroids therapy, surgery, systemic steroids therapy+ antibiotics, and antibiotics, antibiotics+drainage, were ranked lower than observation. This network meta-analysis suggested that three combined treatments (Surgery+Local steroid injection+ Systemic steroids therapy)

have the highest probability of reducing the recurrence rate of IGM. The surface under cumulative ranking curve (SUCRA) values for (Surgery+Local steroid injection+ Systemic steroids therapy) were 0.85, followed by Local steroid injection (SUCRA, 0.78) and (Surgery+Systemic steroids therapy) (SUCRA, 0.77).

Publication bias

The comparison-adjusted funnel plots did not exhibit any signs of apparent asymmetry (Fig. 4). Consequently, no significant publication bias was detected.

Discussion

Several interventions have proven effective in lowering the recurrence rate of idiopathic granulomatous mastitis (IGM). Nonetheless, the existing evidence predominantly derives from direct meta-analyses. Network meta-analysis, a method that allows for the simultaneous comparison of multiple treatments by integrating direct and indirect evidence, provides a more comprehensive evaluation. This study employed a network meta-analysis to scrutinize the available evidence on the effectiveness of different interventions in reducing the recurrence rate among IGM patients.

The results of this network meta-analysis showed that Surgery+Local steroid injection+Systemic steroids therapy has the lowest recurrence rate, followed by Local steroid injection and Surgery+Systemic steroids therapy. In recent years, the strategy of steroid combination with surgical treatment has been gradually applied. Steroid therapy is very effective in the acute phase of IGM, which can significantly relieve local inflammatory symptoms and reduce lesion edema, thereby shrinking the mass. The combination of steroids with surgery leverages the anti-inflammatory effects of

hormones to slow the progression of idiopathic granulomatous mastitis (IGM), restrict the lesion to a smaller area, and preserve more breast tissue, thereby enhancing cosmetic outcomes. Preoperative hormone use aids in clearly delineating the boundaries between diseased and normal breast tissue. This approach facilitates the complete excision of inflammatory tissue and reduces IGM recurrence[36]. In a meta-analysis encompassing 15 different studies, Lei et al. reported recurrence rates for surgical treatment, oral steroids, and combined surgery with oral steroids as 6.8%, 20.9%, and 4%, respectively. The authors concluded that the combined surgery and oral steroid treatment resulted in the lowest recurrence rate[14].

However, the long-term and high-dose use of corticosteroids is associated with a significant risk of adverse effects. Currently, corticosteroids are administered as local injections for the treatment of various inflammatory diseases, and their efficacy is well-documented[37]. Gunduz et al. conducted a pioneering study on the local application of corticosteroids, treating 11 patients with idiopathic granulomatous mastitis (IGM) over a 12-week period. During an average follow-up period of 17 months, two patients experienced a recurrence, and no steroid-related side effects were observed. The success of local corticosteroid application is attributed to its targeted delivery to parenchymal lesions in the breast skin, which is especially beneficial for overweight patients with larger breast tissue and deeper lesion locations[38]. In this study, the recurrence rate for surgery combined with local corticosteroid injections and systemic corticosteroid therapy was lower than that for surgery combined with systemic corticosteroid therapy alone. This suggests that preoperative local corticosteroid

injections, in conjunction with systemic corticosteroid therapy, may enhance therapeutic outcomes.

Akbulut et al. [39] discovered that methotrexate could effectively prevent complications, alleviate the inflammatory process, and reduce the use of hormones through a retrospective analysis of 541 IGM patients. Early immunosuppressive treatment for IGM patients with infection and abscess formation can effectively shorten the course of the disease and reduce recurrence[3, 28]. Kim et al.[9] found that patients who used a combination of methotrexate and steroids were less likely to experience relapse and could maintain remission after discontinuing the medication. It can be concluded that the combined use of hormones and methotrexate may represent a reasonable and effective option for some patients with IGM. It should be noted that the dosage and course of treatment of methotrexate have not been standardized. Simultaneously, folic acid should be taken in combination to prevent folate deficiency syndrome, and attention should also be given to interstitial pneumonia caused by methotrexate. A rigorous evaluation is necessary before use[40].

Drainage requires an extended treatment duration, and the incision is challenging to heal, potentially resulting in the formation of fistulas. Consequently, the utilization of this treatment is a matter of controversy. Erozgen et al.[41] documented 13 patients with IGM and abscesses who underwent incision and drainage. Only 4 of them attained complete relief through drainage; however, the recovery time was prolonged. In the remaining 9 cases, lesions persisted after drainage, necessitating additional steroid therapy. Incision and drainage are options for treating IGM patients with abscesses;

however, it is recommended to combine drainage with other therapeutic approaches.

This study revealed that the recurrence rate associated with observation was surpassed only by combination therapy involving steroids. Some cases of IGM are regarded as self-limiting[42]. Various drug treatment approaches documented in the literature necessitate a treatment duration of at least 6 to 9 months, aligning with the inherent self-healing timeline of the disease. Consequently, discerning whether this reflects the drug's efficacy or the natural course of the disease is challenging. Studies employing close observation and follow-up as the sole intervention reveal that most patients exhibit no evident clinical symptoms. Therefore, close monitoring is an optional nonsurgical treatment for patients diagnosed with GLM at an early stage, exhibiting mild symptoms, who prefer conservative treatment[18].

Given the typical clinical manifestation of IGM as breast inflammation, the majority of patients undergo early antibiotic treatment. However, a significant proportion of IGM patients lack microbiological evidence of bacterial infection, leading to frequent antibiotic treatment failures. Although some researchers have suggested that there may be a link between IGM and Corynebacterium infection, it has not been clear whether the positive result is caused by colonization or primary or secondary infection. Currently, no effective treatment for Corynebacterium exists. Therefore, the existing evidence supporting antibiotic treatment for IGM remains insufficient, and its efficacy is limited[43].

Traditional Chinese medicine is also considered a viable treatment option. The theory of syndrome differentiation and treatment allows for the adjustment of

medications based on the specific symptoms exhibited by the patient. Comprehensive traditional Chinese medicine therapy is associated with a low recurrence rate, minimal tissue damage, and negligible impact on breast appearance[44].

This study has several limitations. Firstly, it did not evaluate the side effects of various interventions due to a lack of corresponding data. Secondly, the included studies had a retrospective design, which introduces potential selection bias. Other limitations include the small sample sizes of some studies and the dosage variations of the same treatment regimen across different centers.

Conclusions

The results of this network meta-analysis suggest that steroid-based combination therapy may be the primary choice for patients with idiopathic granulomatous mastitis (IGM). The combined treatment approach of local and systemic corticosteroids, along with surgery, may represent the most favorable therapeutic strategy for IGM. To support this perspective, additional large-scale randomized controlled trials should be conducted to provide a higher level of evidence.

Reference

1. Benson JR, Dumitru D. Idiopathic granulomatous mastitis: presentation, investigation and management. Future Oncol. 2016;12(11):1381-94. Epub 20160412. doi: 10.2217/fon-2015-0038. PubMed PMID: 27067146.

2. Kessler E, Wolloch Y. Granulomatous mastitis: a lesion clinically simulating carcinoma. Am J Clin Pathol. 1972;58(6):642-6. doi: 10.1093/ajcp/58.6.642. PubMed PMID: 4674439.

3. Uysal E, Soran A, Sezgin E, Granulomatous Mastitis Study G. Factors related to recurrence of idiopathic granulomatous mastitis: what do we learn from a multicentre study? ANZ J Surg. 2018;88(6):635-9. Epub 20170727. doi: 10.1111/ans.14115. PubMed PMID: 28749045.

4. Al-Khaffaf B, Knox F, Bundred NJ. Idiopathic granulomatous mastitis: a 25-year experience. J Am Coll Surg. 2008;206(2):269-73. Epub 20071029. doi: 10.1016/j.jamcollsurg.2007.07.041. PubMed PMID: 18222379.

5. Azlina AF, Ariza Z, Arni T, Hisham AN. Chronic granulomatous mastitis: diagnostic and therapeutic considerations. World J Surg. 2003;27(5):515-8. Epub 20030428. doi: 10.1007/s00268-003-6806-1. PubMed PMID: 12715214.

6. Fruchter R, Castilla C, Ng E, Pomeranz MK, Femia AN. Erythema nodosum in association with idiopathic granulomatous mastitis: a case series and review of the literature. J Eur Acad Dermatol Venereol. 2017;31(9):e391-e3. Epub 20170405. doi: 10.1111/jdv.14194. PubMed PMID: 28271562.

7. Renshaw AA, Derhagopian RP, Gould EW. Cystic neutrophilic granulomatous mastitis: an underappreciated pattern strongly associated with gram-positive bacilli. Am J Clin Pathol. 2011;136(3):424-7. doi: 10.1309/AJCP1W9JBRYOQSNZ. PubMed PMID: 21846918.

8. Schelfout K, Tjalma WA, Cooremans ID, Coeman DC, Colpaert CG, Buytaert PM. Observations of an idiopathic granulomatous mastitis. Eur J Obstet Gynecol Reprod Biol. 2001;97(2):260-2. doi: 10.1016/s0301-2115(00)00546-7. PubMed PMID: 11451563.

9. Kim J, Tymms KE, Buckingham JM. Methotrexate in the management of granulomatous mastitis. ANZ J Surg. 2003;73(4):247-9. doi: 10.1046/j.1445-1433.2002.02564.x. PubMed PMID: 12662235.

10. Maffini F, Baldini F, Bassi F, Luini A, Viale G. Systemic therapy as a first choice treatment for idiopathic granulomatous mastitis. J Cutan Pathol. 2009;36(6):689-91. Epub 20090204. doi: 10.1111/j.1600-0560.2008.01102.x. PubMed PMID: 19222698.

11. Schmajuk G, Genovese MC. First report of idiopathic granulomatous mastitis treated with methotrexate monotherapy. J Rheumatol. 2009;36(7):1559-60. doi: 10.3899/jrheum.090091. PubMed PMID: 19567642.

12. Patel RA, Strickland P, Sankara IR, Pinkston G, Many W, Jr., Rodriguez M. Idiopathic granulomatous mastitis: case reports and review of literature. J Gen Intern Med. 2010;25(3):270-3. Epub 20091215. doi: 10.1007/s11606-009-1207-2. PubMed PMID: 20013067; PubMed Central PMCID: PMCPMC2839326.

13. Godazandeh G, Shojaee L, Alizadeh-Navaei R, Hessami A. Corticosteroids in idiopathic granulomatous mastitis: a systematic review and meta-analysis. Surg Today. 2021;51(12):1897-905. Epub 20210215. doi: 10.1007/s00595-021-02234-4. PubMed PMID: 33590327.

14. Lei X, Chen K, Zhu L, Song E, Su F, Li S. Treatments for Idiopathic Granulomatous Mastitis: Systematic Review and Meta-Analysis. Breastfeed Med. 2017;12(7):415-21. Epub 20170721. doi: 10.1089/bfm.2017.0030. PubMed PMID: 28731822.

15. Lumley T. Network meta-analysis for indirect treatment comparisons. Stat Med. 2002;21(16):2313-24. doi: 10.1002/sim.1201. PubMed PMID: 12210616.

16. Rouse B, Chaimani A, Li T. Network meta-analysis: an introduction for clinicians. Intern Emerg Med. 2017;12(1):103-11. Epub 20161202. doi: 10.1007/s11739-016-1583-7. PubMed PMID: 27913917; PubMed Central PMCID: PMCPMC5247317.
17. Akahane K, Tsunoda N, Kato M, Noda S, Shimoyama Y, Ishigakis S, et al. Therapeutic strategy for granulomatous lobular mastitis: a clinicopathological study of 12 patients. Nagoya J Med Sci. 2013;75(3-4):193-200. PubMed PMID: 24640175; PubMed Central PMCID: PMCPMC4345679.
18. Lai EC, Chan WC, Ma TK, Tang AP, Poon CS, Leong HT. The role of conservative treatment in idiopathic granulomatous mastitis. Breast J. 2005;11(6):454-6. doi: 10.1111/j.1075-122X.2005.00127.x. PubMed PMID: 16297091.
19. Skandarajah A, Marley L. Idiopathic granulomatous mastitis: a medical or surgical disease of the breast? ANZ J Surg. 2015;85(12):979-82. Epub 20141126. doi: 10.1111/ans.12929. PubMed PMID: 25424519.
20. Shin YD, Park SS, Song YJ, Son SM, Choi YJ. Is surgical excision necessary for the treatment of Granulomatous lobular mastitis? BMC Womens Health. 2017;17(1):49. Epub 20170724. doi: 10.1186/s12905-017-0412-0. PubMed PMID: 28738795; PubMed Central PMCID: PMCPMC5525244.
21. Sheybani F, Sarvghad M, Naderi H, Gharib M. Treatment for and clinical characteristics of granulomatous mastitis. Obstet Gynecol. 2015;125(4):801-7. doi: 10.1097/AOG.0000000000000734. PubMed PMID: 25751209.
22. Sakurai K, Fujisaki S, Enomoto K, Amano S, Sugitani M. Evaluation of follow-up strategies for corticosteroid therapy of idiopathic granulomatous mastitis. Surg Today. 2011;41(3):333-7. Epub 20110302. doi: 10.1007/s00595-009-4292-2. PubMed PMID: 21365412.
23. Oran ES, Gurdal SO, Yankol Y, Oznur M, Calay Z, Tunaci M, et al. Management of idiopathic granulomatous mastitis diagnosed by core biopsy: a retrospective multicenter study. Breast J. 2013;19(4):411-8. Epub 20130512. doi: 10.1111/tbj.12123. PubMed PMID: 23663101.
24. Ren Y, Zhang J, Zhang J, Guo R. Combining intralesional steroid injection with oral steroids in patients with idiopathic granulomatous mastitis. Medicine (Baltimore). 2023;102(24):e34055. doi: 10.1097/MD.0000000000034055. PubMed PMID: 37327292; PubMed Central PMCID: PMCPMC10270520.
25. Zhang X, Li J, Hu XJ. Postoperative Yanghe decoction regimen improves outcomes for idiopathic granulomatous mastitis: A retrospective cohort study. Medicine (Baltimore). 2020;99(45):e23136. doi: 10.1097/MD.0000000000023136. PubMed PMID: 33157994; PubMed Central PMCID: PMCPMC7647528.
26. Liu PZ, Shi XG, Zuo XM, Lai R, Zhao Z, Yang ZR, et al. A clinical study on the treatment of granulomatous lobular mastitis by the external application of the internal pus-expelling decoction and operation. Ann Palliat Med. 2020;9(5):2631-41. Epub 20200825. doi: 10.21037/apm-19-684. PubMed PMID: 32921065.
27. Karanlik H, Ozgur I, Simsek S, Fathalizadeh A, Tukenmez M, Sahin D, et al. Can Steroids plus Surgery Become a First-Line Treatment of Idiopathic Granulomatous Mastitis? Breast Care (Basel). 2014;9(5):338-42. doi: 10.1159/000366437. PubMed PMID: 25759614; PubMed Central PMCID: PMCPMC4322692.
28. Hur SM, Cho DH, Lee SK, Choi MY, Bae SY, Koo MY, et al. Experience of treatment of patients with granulomatous lobular mastitis. J Korean Surg Soc. 2013;85(1):1-6. Epub 20130626. doi: 10.4174/jkss.2013.85.1.1. PubMed PMID: 23833753; PubMed Central PMCID: PMCPMC3699681.
29. Akcan A, Oz AB, Dogan S, Akgun H, Akyuz M, Ok E, et al. Idiopathic Granulomatous Mastitis:

Comparison of Wide Local Excision with or without Corticosteroid Therapy. Breast Care (Basel). 2014;9(2):111-5. doi: 10.1159/000360926. PubMed PMID: 24944554; PubMed Central PMCID: PMCPMC4038310.
30. Shojaee L, Rahmani N, Moradi S, Motamedi A, Godazandeh G. Idiopathic granulomatous mastitis: challenges of treatment in iranian women. BMC Surg. 2021;21(1):206. Epub 20210421. doi: 10.1186/s12893-021-01210-6. PubMed PMID: 33882924; PubMed Central PMCID: PMCPMC8061012.
31. Chirappapha P, Thaweepworadej P, Supsamutchai C, Biadul N, Lertsithichai P. Idiopathic granulomatous mastitis: A retrospective cohort study between 44 patients with different treatment modalities. Ann Med Surg (Lond). 2018;36:162-7. Epub 20181109. doi: 10.1016/j.amsu.2018.11.001. PubMed PMID: 30479764; PubMed Central PMCID: PMCPMC6240599.
32. Yabanoglu H, Colakoglu T, Belli S, Aytac HO, Bolat FA, Pourbagher A, et al. A Comparative Study of Conservative versus Surgical Treatment Protocols for 77 Patients with Idiopathic Granulomatous Mastitis. Breast J. 2015;21(4):363-9. Epub 20150410. doi: 10.1111/tbj.12415. PubMed PMID: 25858348.
33. Erturk TF, Cakir O, Yaprak Bayrak B, Gunes A, Aydemir S, Utkan NZ. Local Steroid Treatment: An Effective Procedure for Idiopathic Granulomatous Mastitis, Including Complicated Cases. J Invest Surg. 2022;35(4):745-51. Epub 20210621. doi: 10.1080/08941939.2021.1933272. PubMed PMID: 34154493.
34. Wang J, Zhang Y, Lu X, Xi C, Yu K, Gao R, et al. Idiopathic Granulomatous Mastitis with Skin Rupture: A Retrospective Cohort Study of 200 Patients Who Underwent Surgical and Nonsurgical Treatment. J Invest Surg. 2021;34(7):810-5. Epub 20191210. doi: 10.1080/08941939.2019.1696905. PubMed PMID: 31818161.
35. Alper F, Karadeniz E, Guven F, Cankaya BY, Yalcin A, Ozden K, et al. Comparison of the Efficacy of Systemic Versus Local Steroid Treatment in Idiopathic Granulomatous Mastitis: A Cohort Study. J Surg Res. 2022;278:86-92. Epub 20220517. doi: 10.1016/j.jss.2022.04.015. PubMed PMID: 35594619.
36. Wang C, Lin Y, Zhou Y, Sun Q. Novel Paradigm for Treating Idiopathic Granulomatous Mastitis. J Invest Surg. 2021;34(7):816-7. Epub 20200121. doi: 10.1080/08941939.2019.1711269. PubMed PMID: 31960715.
37. Konai MS, Vilar Furtado RN, Dos Santos MF, Natour J. Monoarticular corticosteroid injection versus systemic administration in the treatment of rheumatoid arthritis patients: a randomized double-blind controlled study. Clin Exp Rheumatol. 2009;27(2):214-21. PubMed PMID: 19473560.
38. Gunduz Y, Altintoprak F, Tatli Ayhan L, Kivilcim T, Celebi F. Effect of topical steroid treatment on idiopathic granulomatous mastitis: clinical and radiologic evaluation. Breast J. 2014;20(6):586-91. Epub 20140917. doi: 10.1111/tbj.12335. PubMed PMID: 25228089.
39. Akbulut S, Arikanoglu Z, Senol A, Sogutcu N, Basbug M, Yeniaras E, et al. Is methotrexate an acceptable treatment in the management of idiopathic granulomatous mastitis? Arch Gynecol Obstet. 2011;284(5):1189-95. Epub 20110105. doi: 10.1007/s00404-010-1825-2. PubMed PMID: 21207047.
40. Lermi N, Ekin A, Ocak T, Bozkurt ZY, Otegeceli MA, Yagiz B, et al. What predicts the recurrence in idiopathic granulomatous mastitis? Clin Rheumatol. 2023;42(9):2491-500. Epub 20230610. doi: 10.1007/s10067-023-06651-3. PubMed PMID: 37301771.
41. Erozgen F, Ersoy YE, Akaydin M, Memmi N, Celik AS, Celebi F, et al. Corticosteroid treatment and timing of surgery in idiopathic granulomatous mastitis confusing with breast carcinoma. Breast Cancer Res Treat. 2010;123(2):447-52. Epub 20100713. doi: 10.1007/s10549-010-1041-6. PubMed PMID: 20625813.
42. Fattahi AS, Amini G, Sajedi F, Mehrad-Majd H. Factors Affecting Recurrence of Idiopathic

Granulomatous Mastitis: A Systematic Review. Breast J. 2023;2023:9947797. Epub 20230926. doi: 10.1155/2023/9947797. PubMed PMID: 37794976; PubMed Central PMCID: PMCPMC10547579.
43. Co M, Cheng VCC, Wei J, Wong SCY, Chan SMS, Shek T, et al. Idiopathic granulomatous mastitis: a 10-year study from a multicentre clinical database. Pathology. 2018;50(7):742-7. Epub 20181030. doi: 10.1016/j.pathol.2018.08.010. PubMed PMID: 30389215.
44. Zuo X, Shi X, Gao X, Lai R, Liu P, Zhao Z. A retrospective study on 221 patients with granulomatous lobular mastitis treated by a combination of traditional Chinese medicine and western medicine. Ann Ital Chir. 2021;92:135-41. PubMed PMID: 34031280.

 Click here to access/download;Figure;Fig.docx

Fig. 1 Flowchart of the study.

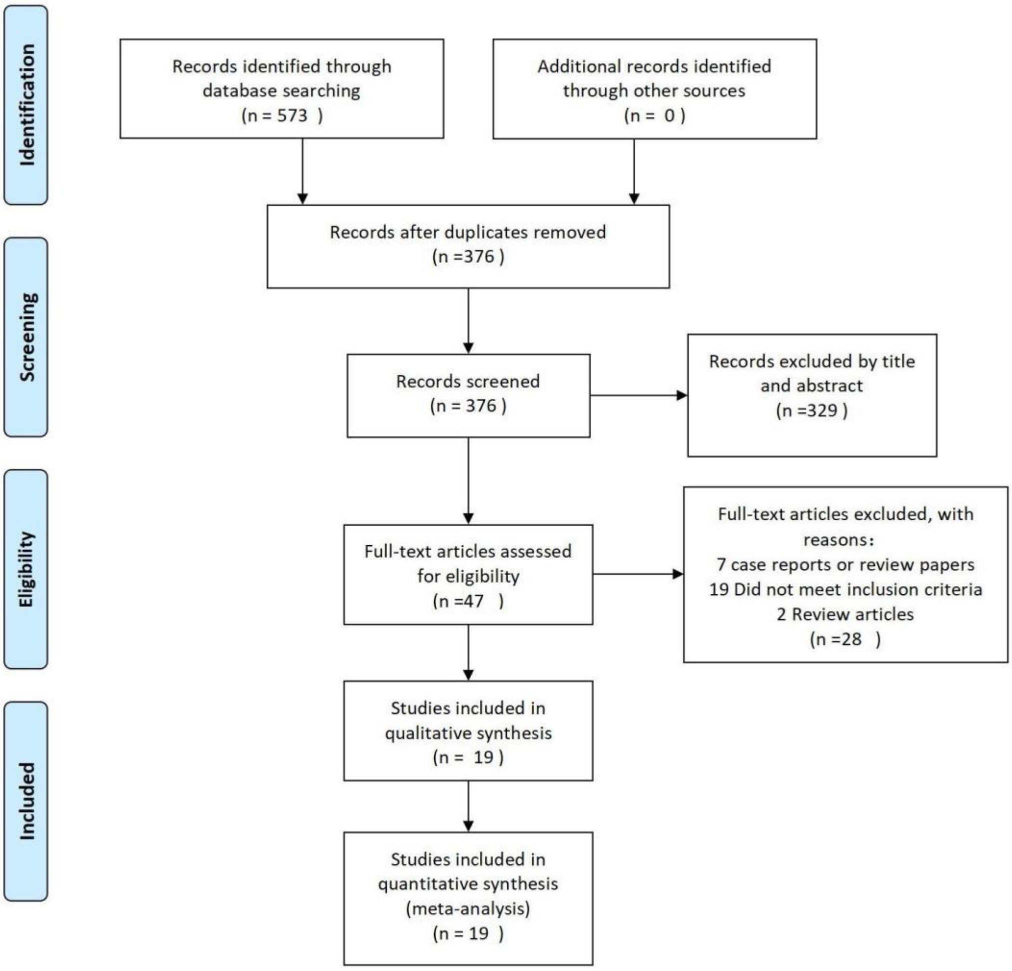

Fig. 2 Network plot of Granulomatous lobular mastitis in various treatments

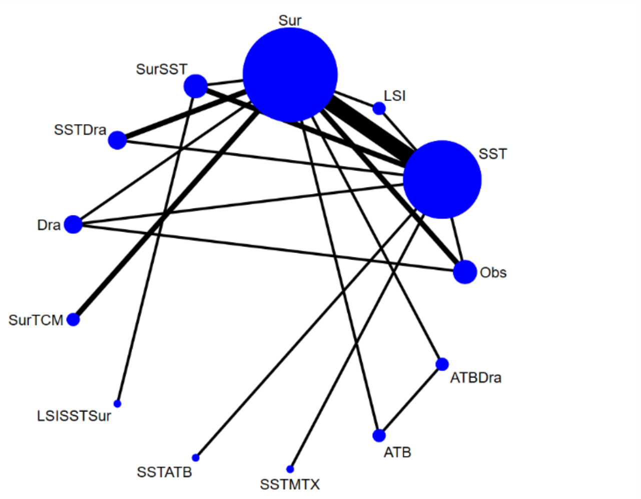

Fig 3. Rankograms for the network show the probability of the recurrence rate of different treatments for IGM reduced. Observation, Obs; Systemic steroids therapy, SST; Local steroid injection, LSI; Surgery, Sur; Surgery+Systemic steroids therapy, SurSST; Systemic steroids therapy+ drainage, SSTDra; drainage, Dra; Surgery+ Traditional Chinese Medicine, SurTCM; Surgery+Local steroid injection+ Systemic steroids therapy, LSISSTSur; Systemic steroids therapy+Antibiotics, SSTATB; Systemic steroids therapy+ MTX, SSTMTX; Antibiotics, ATB; Antibiotics+drainage, ATBDra.

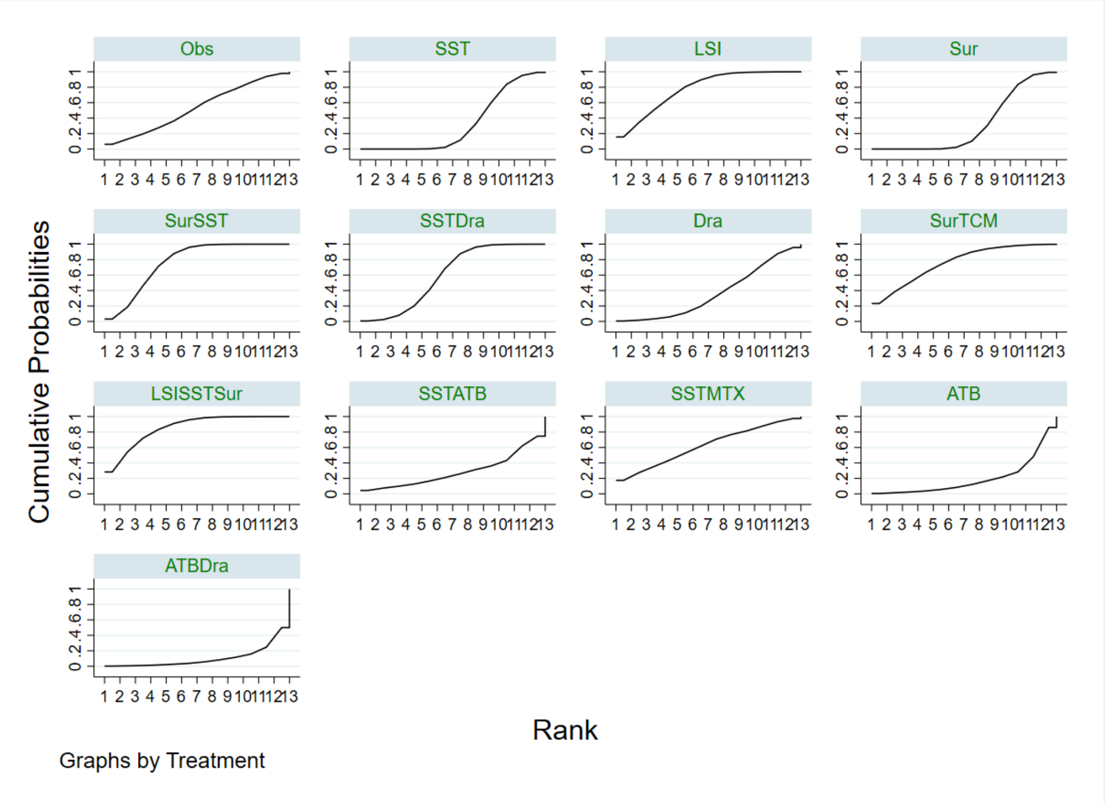

Fig 4. Comparison-adjusted funnel plot for the selected studies with the recurrence rate of different treatments for IGM. Abbreviation: A, Observation; B, Systemic steroids therapy; C, Local steroid injection;D, Surgery; E, Surgery+Systemic steroids therapy; F, Systemic steroids therapy+ drainage; G, Drainage; H, Surgery+Traditional Chinese Medicine; I, Surgery+Local steroid injection+ Systemic steroids therapy; J, Systemic steroids therapy+Antibiotics; K, Systemic steroids therapy+MTX; L, Antibiotics; M, Antibiotics+drainage.

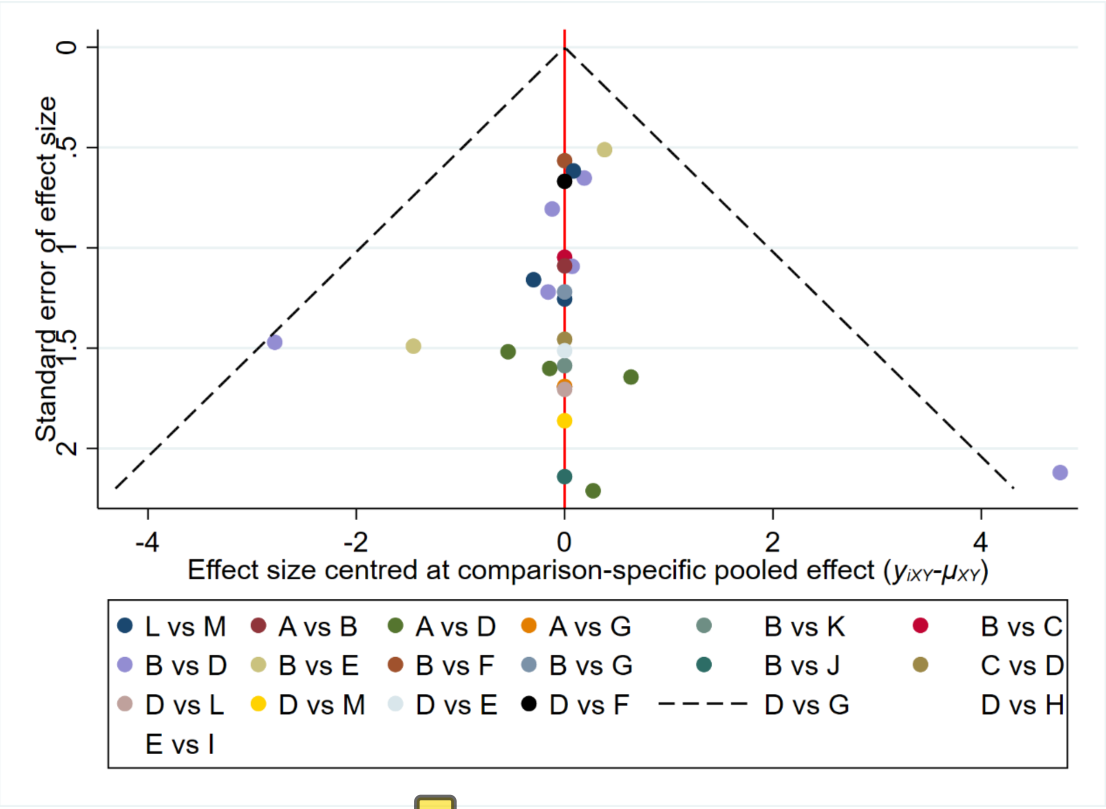

 Click here to access/download;Figure;Table 1.docx

Table 1. Characteristics of included studies.

**Study**	**Intervention**	**Sample size**	**Total recurrence**	**Follow-up**	**Quality assessment**	**Type of study**
Fatih 2022	Systemic steroids therapy	16	2	1-5 months	8/9	Cohort
	Local steroid injection	42	2	1-12months		
Jiang 2019	Systemic steroids therapy	44	10	12-36 months	8/9	Cohort
	Surgery+Systemic steroids therapy	156	8			
Taha 2021	Local steroid injection	38	0	12 months	7/9	Cohort
	Surgery	48	15			
Hakan 2015	Systemic steroids therapy	44	9	1-38 months	8/9	Cohort
	Surgery	33	0	1-120 months		
Kazuhisa 2013	Systemic steroids therapy	10	0	6-104 months	7/9	Cohort
	Surgery	2	2	26-77 monyhs		

Prakasit2018	Systemic steroids therapy	6	1	5-12 months	7/9	Cohort
	Surgery	30	4			
Leyla 2021	Systemic steroids therapy	23	9	8-48 months	7/9	Cohort
	Surgery	17	7			
	Systemic steroids therapy+ drainage	47	9			
Alper 2014	Surgery	53	4	3-170 months	6/9	Cohort
	Surgery+Systemic steroids therapy	21	0			
Sung 2013	Observation	8	0	14-40 months	6/9	Cohort
	Systemic steroids therapy	13	1			
	Surgery	23	2			
	Drainage	14	1			
Hasan 2014	Systemic steroids therapy	23	7	2-18 months	7/9	Cohort
	Surgery+Systemic steroids therapy	37	0	22-78 months		
Lai 2005	Observation	4	0	1-24 months	6/9	Cohort

	Surgery	1	0	1-30 months		
Liu 2020	Surgery	50	1	1-12 months	7/9	Cohort
	Surgery+Traditional Chinese Medicine	60	0			
Zhang2020	Surgery	25	4	3-23 months	7/9	Cohort
	Surgery+Traditional Chinese Medicine	28	0	2-22 months		
Ren 2013	Surgery+Systemic steroids therapy	28	6	24 months	7/9	Cohort
	Surgery+Local steroid injection+ Systemic steroids therapy	34	5			
Oran2013	Systemic steroids therapy	25	5	3-135 months	7/9	Cohort
	Surgery	18	3			
SAKURAI 2011	Systemic steroids therapy	5	0	1–39 months	6/9	Cohort
	Systemic steroids therapy+Antibiotics	2	0			
Sheybani 2015	Systemic steroids	15	3	6-22 months	7/9	Cohort

	therapy					
	Systemic steroids therapy+MTX	6	0			
Shin2017	Surgery	20	5	22-98 months	8/9	Cohort
	Systemic steroids therapy+ drainage	14	1			
Skandarajah2014	Surgery	2	0	0-24 monhs	6/9	Cohort
	Antibiotics	7	3			
	Antibiotics+drainage	3	2			

Table 2. Results of the recurrence rate of different treatments for IGM reduced; results presented as constant odds ratios between all competing interventions with 95% confidence intervals. Observation, Obs; Systemic steroids therapy, SST; Local steroid injection, LSI; Surgery, Sur; Surgery+Systemic steroids therapy, SurSST; Systemic steroids therapy+ drainage, SSTDra; drainage, Dra; Surgery+ Traditional Chinese Medicine, SurTCM; Surgery+Local steroid injection+ Systemic steroids therapy, LSISSTSur; Systemic steroids therapy+Antibiotics, SSTATB; Systemic steroids therapy+ MTX, SSTMTX; Antibiotics, ATB; Antibiotics+drainage, ATBDra.

LSISSTSur												
0.68 (0.07,6.82)	LSI											
0.63 (0.17,2.34)	0.93 (0.14,6.27)	SurSST										
0.69 (0.04,11.14)	1.02 (0.06,16.57)	1.09 (0.09,12.71)	SurTCM									
0.37 (0.01,12.07)	0.54 (0.02,18.64)	0.58 (0.02,14.80)	0.53 (0.01,25.65)	SSTMTX								
0.30 (0.05,1.91)	0.44 (0.07,2.95)	0.48 (0.13,1.75)	0.44 (0.04,4.75)	0.82 (0.03,21.29)	SSTDra							
0.23 (0.01,4.53)	0.34 (0.02,6.81)	0.36 (0.02,5.29)	0.33 (0.01,9.11)	0.62 (0.01,34.59)	0.76 (0.05,10.67)	Obs						
0.11 (0.01,1.39)	0.16 (0.01,2.11)	0.17 (0.02,1.54)	0.16 (0.01,2.99)	0.29 (0.01,11.98)	0.36 (0.04,3.10)	0.47 (0.03,8.88)	Dra					
0.10 (0.02,0.49)	0.15 (0.03,0.80)	0.16 (0.06,0.39)	0.15 (0.01,1.47)	0.27 (0.01,6.17)	0.34 (0.13,0.88)	0.44 (0.03,5.65)	0.94 (0.12,7.12)	SST				
0.10 (0.02,0.56)	0.15 (0.03,0.83)	0.16 (0.05,0.48)	0.14 (0.02,1.28)	0.27 (0.01,6.62)	0.33 (0.13,0.86)	0.44 (0.04,5.27)	0.92 (0.13,6.74)	0.98 (0.47,2.06)	Sur			
0.05 (0.00,4.07)	0.07 (0.00,6.22)	0.07 (0.00,5.29)	0.07 (0.00,8.00)	0.12 (0.00,23.16)	0.15 (0.00,11.29)	0.20 (0.00,27.23)	0.43 (0.00,45.03)	0.45 (0.01,30.17)	0.46 (0.01,32.69)	SSTATB		
0.03 (0.00,1.10)	0.04 (0.00,1.63)	0.04 (0.00,1.37)	0.04 (0.00,2.01)	0.07 (0.00,7.10)	0.08 (0.00,2.75)	0.11 (0.00,7.25)	0.24 (0.00,11.61)	0.25 (0.01,7.77)	0.26 (0.01,7.27)	0.56 (0.00,125.17)	ATB	
0.01 (0.00,0.67)	0.02 (0.00,1.00)	0.02 (0.00,0.86)	0.02 (0.00,1.22)	0.03 (0.00,4.16)	0.04 (0.00,1.72)	0.05 (0.00,4.34)	0.11 (0.00,7.08)	0.12 (0.00,4.89)	0.12 (0.00,4.61)	0.26 (0.00,70.97)	0.47 (0.04,5.46)	ATBDra

Table 3.Treatment Relative Ranking

Treatment	SUCRA	PrBest	MeanRank
LSISSTSur	85.2	28.3	2.8
LSI	77.5	15.6	3.7
SurSST	76.9	3.1	3.8
SurTCM	75.9	23.2	3.9
SSTMTX	62.2	17.4	5.5
SSTDra	60.3	0.6	5.8
Obs	53.1	6.1	6.6
Dra	36.2	0.6	8.7
SST	32.1	0	9.1
Sur	31.7	0	9.2
SSTATB	28.8	4.4	9.5
ATB	19.6	0.5	10.6
ATBDra	10.5	0.2	11.7

Guideline Flow Chart

Click here to access/download
Supporting Information
OA_Guidelines Flow Chart.jpg

Click here to access/download
Supporting Information
OA_Guidelines checklist.docx
